# Role of Hydrogen Sulfide in Ischemia-Reperfusion Injury

**DOI:** 10.1155/2015/186908

**Published:** 2015-05-12

**Authors:** Dongdong Wu, Jun Wang, Hui Li, Mengzhou Xue, Ailing Ji, Yanzhang Li

**Affiliations:** ^1^Medical College of Henan University, Kaifeng, Henan 475004, China; ^2^Department of Neurology, Institute of Neurological Disorders, The First Affiliated Hospital of Henan University, Kaifeng 475001, China

## Abstract

Ischemia-reperfusion (I/R) injury is one of the major causes of high morbidity, disability, and mortality in the world. I/R injury remains a complicated and unresolved situation in clinical practice, especially in the field of solid organ transplantation. Hydrogen sulfide (H_2_S) is the third gaseous signaling molecule and plays a broad range of physiological and pathophysiological roles in mammals. H_2_S could protect against I/R injury in many organs and tissues, such as heart, liver, kidney, brain, intestine, stomach, hind-limb, lung, and retina. The goal of this review is to highlight recent findings regarding the role of H_2_S in I/R injury. In this review, we present the production and metabolism of H_2_S and further discuss the effect and mechanism of H_2_S in I/R injury.

## 1. Introduction

Ischemia-reperfusion (I/R) is a well-recognized pathological condition that is characterized by an initial deprivation of blood supply to an area or organ followed by subsequent vascular restoration and concomitant reoxygenation of downstream tissue [1]. I/R can develop as a consequence of trauma, hypertension, shock, sepsis, organ transplantation, or bypass surgery leading to end-organ failure such as acute renal tubular necrosis, bowel infarct, and liver failure. I/R can also occur under various complications of vascular diseases such as stroke and myocardial infarction [1, 2]. Several pathophysiologic mechanisms have been proposed as mediators of the damage induced by I/R, such as activation of the complement system and leukocyte recruitment, endoplasmic reticulum stress, calcium overload, reduction of oxidative phosphorylation, increased free radical concentration, development of the no-reflow phenomenon, endothelial dysfunction, and activation of signaling pathways of apoptosis, necrosis, and/or autophagy [1, 3]. Many studies have shown that there are three time frames in the protection against I/R injury: before the index ischemic episode (ischemic preconditioning), during ischemia (ischemic conditioning), and at the onset of reperfusion (ischemic postconditioning) [4, 5]. Currently, several therapeutic gases have been shown to play a role in the treatment of I/R injury, including hydrogen, nitric oxide (NO), carbon monoxide (CO), and hydrogen sulfide (H_2_S) [6].

H_2_S is a colorless, flammable, and water-soluble gas with the characteristic smell of rotten eggs. In the past several centuries, H_2_S had been known only for its toxicity and environmental hazards [7, 8]. It elicits its toxic effects by reversibly inhibiting cytochrome c oxidase (CcO), preventing oxidative phosphorylation and lowering the production of adenosine triphosphate (ATP). Recently, there has been growing evidence that H_2_S plays a broad range of physiological and pathophysiological functions [9, 10], including induction of angiogenesis [11], regulation of neuronal activity [9], vascular relaxation [12], glucose homeostatic regulation [13], and protection against I/R injury in heart, liver, kidney, lung, and brain [14–18]. The abnormal metabolism of H_2_S could result in an array of pathological disturbances in the form of hypertension, diabetes, atherosclerosis, heart failure, sepsis, inflammation, erectile dysfunction, cataracts, asthma, and neurodegenerative diseases [10]. In addition, H_2_S can also interact with other specific molecules, including NO [19], CcO [20], catalase [21], myoglobin [21, 22], hemoglobin [21, 22], Kelch-like ECH-associated protein 1 (Keap1) [23], cysteine residues on ATP-sensitive potassium (K_ATP_) channels [24], epidermal growth factor receptor [25], and vascular endothelial growth factor receptor 2 [25, 26]. Considering H_2_S is involved in numerous biological processes, it is now widely accepted that H_2_S functions as the third signaling gasotransmitter, along with NO and CO [9].

With the deepening of research on H_2_S and I/R injury, the role that H_2_S plays in attenuating I/R injury has begun to be elucidated. In this review, we highlight recent studies that provide new insight into the production and metabolism of H_2_S and discuss the role and mechanism of H_2_S on I/R injury.

## 2. Production and Metabolism of **H_2_**S

### 2.1. Endogenous Production of H_2_S

H_2_S is endogenously generated in mammalian cells via both enzymatic and nonenzymatic pathways, although the nonenzymatic pathway is less important in H_2_S production [27]. With regard to the enzymatic pathway, cystathionine *β*-synthase (CBS) and cystathionine *γ*-lyase (CSE) are two pyridoxal-5′-phosphate- (PLP-) dependent enzymes, which use either L-cysteine or L-cysteine together with homocysteine as their principal substrates to produce H_2_S [9]. Unlike CBS and CSE, 3-mercaptopyruvate sulfurtransferase (3-MST) is a PLP-independent enzyme, which uses 3-mercaptopyruvate (3MP) as a substrate to produce H_2_S. 3MP is a metabolite of L-cysteine and *α*-ketoglutarate by cysteine aminotransferase (CAT) [9]. CSE and CBS are cytosolic enzymes with tissue-specific distributions. CBS is predominantly expressed in the central nervous system and is also found in liver, kidney, ileum, uterus, placenta, and pancreatic islets. CSE is abundant in heart, liver, kidney, uterus, ileum, placenta, and vascular smooth muscle. CSE is the most relevant H_2_S-producing enzyme in the cardiovascular system [9, 27]. CAT and 3-MST are localized both in cytosol and mitochondria, but the majority of these two enzymes are present in the mitochondria [9]. They have been found in the heart, kidney, liver, lung, thymus, testis, brain, and thoracic aorta and are apparently important for H_2_S production in the brain and vasculature [9, 27, 28]. Furthermore, a recent study has demonstrated that D-cysteine (a negative control of L-cysteine) can be metabolized to achiral 3MP by D-amino acid oxidase and can be used as a substrate for 3-MST to produce H_2_S in both kidney and brain [29]. During the enzymatic pathway, H_2_S can be immediately released or stored in a form of bound or acid-labile sulfur in the cells [30].

Apart from enzymatic pathway, endogenous H_2_S can also be produced through nonenzymatic processes that are less well understood [27, 30, 31]. Nonenzymatic production of H_2_S occurs through glucose, inorganic, and organic polysulfides (present in garlic), glutathione, and elemental sulfur [30, 31]. H_2_S can be generated from glucose either via glycolysis (>90%) or from phosphogluconate via nicotinamide adenine dinucleotide phosphate (NADPH) oxidase (<10%) [7, 27, 30]. Glucose could react with cysteine, methionine, or homocysteine to produce gaseous sulfur compounds such as H_2_S and methanethiol [7, 8, 30]. H_2_S is also produced through direct reduction of glutathione and elemental sulfur. Reduction of elemental sulfur to H_2_S is mediated through reducing equivalents of the glucose oxidation pathways such as nicotinamide adenine dinucleotide and NADPH [7, 8]. Thiosulfate is an intermediate of sulfur metabolism from cysteine and H_2_S formation from thiosulfate through a reductive reaction involving pyruvate, which acts as a hydrogen donor [7, 8, 32, 33]. In addition, garlic and garlic-derived organic polysulfides could induce H_2_S production in a thiol-dependent manner, such as diallyl sulfide (DAS), diallyl disulfide (DADS), diallyl trisulfide (DATS), and S-allyl cysteine (SAC) [30–34].

### 2.2. Exogenous Source of H_2_S

H_2_S gas has been considered as the authentic resource of exogenous H_2_S [35]. Recent studies have shown that H_2_S gas plays important roles in promoting angiogenesis [11], ameliorating type II diabetes [13], and protecting against myocardial I/R injury [36]. However, H_2_S gas is not an ideal resource due to a possible toxic impact of H_2_S excess and difficulty in obtaining precisely controlled concentration [35]. Currently, a number of H_2_S-releasing compounds have already been successfully designed and developed. These compounds could be mainly divided into two types, including the “H_2_S donors,” which release H_2_S as the only mechanism of action, and the “H_2_S-releasing hybrid drugs,” also known as “dirty drugs” in which H_2_S release is an ancillary property which accompanies a principal mechanism of the hybrid drugs [35]. Inorganic sulfide salts, such as sodium hydrosulfide (NaHS), sodium sulfide (Na_2_S), and calcium sulfide, have been widely used as H_2_S donors [7, 8, 35]. As the maximum concentration of H_2_S released from these salts can be reached within seconds, they have been called fast-releasing H_2_S donors [35]. However, the effective residence time of these donors in tissues may be very short because H_2_S is highly volatile in solutions [35]. Ideal H_2_S donors for therapeutic purposes should generate H_2_S with relatively slow-releasing rates and longer periods of treating time. Recently, many slow-releasing H_2_S donors (Table 1) and H_2_S-releasing hybrid drugs (Table 2) have been designed and synthesized to increase the treatment efficacy of H_2_S.

### 2.3. Metabolism of H_2_S

In order to maintain a proper physiological balance of its metabolism, H_2_S can be broken down through several enzymatic and nonenzymatic processes [7, 10, 37]. The main pathway of H_2_S catabolism occurs in mitochondria. Mitochondrial oxidative modification converts H_2_S into thiosulfate through several enzymes including quinone oxidoreductase, S-dioxygenase, and S-transferase. Thiosulfate could be further converted into sulfite, which is catalyzed by thiosulfate : cyanide sulfurtransferase. Sulfite is then rapidly oxidized to sulfate by sulfite oxidase. Therefore, sulfate is a major end-product of H_2_S metabolism under physiological conditions [7, 10, 37, 38]. The secondary mechanism of H_2_S catabolism is the methylation to methanethiol and dimethylsulfide via thiol S-methyltransferase in the cytosol [10, 37, 38]. The third pathway of H_2_S metabolism is the interaction of H_2_S with methemoglobin that leads to sulfhemoglobin, which is considered as a possible biomarker of plasma H_2_S [10, 37, 38]. These three pathways are considered the main processes of H_2_S catabolism in mammals. Furthermore, recent studies have shown that H_2_S could be converted into sulfite via minor oxidative routes in activated neutrophils [10, 37].

## 3. **H_2_**S and I/R Injury

### 3.1. H_2_S and Myocardial I/R Injury

Myocardial ischemia is a common clinical symptom characterized by low pH values, low oxygen, and high extracellular potassium concentration, which may cause arrhythmias, cardiac dysfunction, myocardial infarction, and sudden death [3, 5, 6]. The damaged myocardial structure and decreased heart function induced by ischemia can be repaired with subsequent reperfusion. The effectiveness of reperfusion depends on the duration and severity of prior ischemia [6, 39]. However, myocardial reperfusion could also activate a complex inflammatory response, which may finally lead to myocardial ischemia/reperfusion injury (MIRI), such as arrhythmias, myocardial stunning, microvascular dysfunction, and myocyte death [2, 40]. Therefore, it is necessary to develop effective cardioprotective strategies and agents against MIRI to improve myocardial function and to reduce the risk of cardiovascular events [4]. H_2_S is now considered as an endogenous signaling molecule which plays an important role in the cardiovascular system [6, 15, 27]. In the heart, H_2_S is produced in the fibroblasts, myocardium, and blood vessels from L-cysteine by CSE, CBS, and 3-MST and accumulates at relatively high local concentrations [6, 27, 30]. An accumulating body of evidence indicates that exogenous or endogenous H_2_S could exert cardioprotection against MIRI in cardiac myocytes, isolated hearts, and intact animals. However, it is currently difficult to define the precise underlying mechanisms for this protection. A summary of what is known about the mechanisms by which H_2_S and its donors-induced cardioprotection against MIRI is shown in Table 3.

### 3.2. H_2_S and Hepatic I/R Injury

Liver I/R-induced injury represents a continuum of organic processes that could produce profound liver damage and ultimately lead to morbidity and mortality [41, 42]. Hepatic I/R injury has now been considered a worldwide health problem and usually occurs in liver transplantation, hemorrhagic shock and resuscitation, trauma, liver resection surgery, and aortic injury during abdominal surgery [41–43]. Hepatic I/R injury can be categorized into warm I/R and cold storage reperfusion injury, which share a common mechanism in the disease aetiology [41, 42]. Increasing number of experimental and clinical studies indicate that pathways/factors involved in the hepatic I/R injury include liver Kupffer cells and neutrophils, intracellular calcium overload, oxidative stress, anaerobic metabolism, mitochondria, adhesion molecules, chemokines, and proinflammatory cytokines [41, 42, 44, 45]. Despite significant advances in surgical techniques and perioperative cares, hepatic I/R injury remains one of the major complications in hepatic resection and transplantation [46]. Novel agents/drugs exhibiting antioxidative, anti-inflammatory, and cytoprotective activities may be possible candidates for protecting the liver from I/R injury [46]. Recent studies have shown that H_2_S could significantly attenuate hepatic I/R injury in several ways, including inflammation, apoptosis, oxidation, and AKT activation (Table 4). The results suggest that H_2_S has a protective effect against hepatic I/R injury, and targeting H_2_S may present a promising approach against I/R-induced liver injury.

### 3.3. H_2_S and Renal I/R Injury

Acute kidney injury (AKI) is a common and serious complication of critical illness and is associated with high morbidity, mortality, and resource utilization [25, 47, 48]. Renal I/R injury is one of the leading causes of AKI in many clinical settings [47, 48]. Renal I/R injury often arises from shock and various surgical procedures such as kidney transplantation and resection [47–49]. H_2_S plays important physiological and pathological roles in the kidney [48]. For instance, it participates in the control of renal function and increases urinary sodium excretion via both tubular and vascular actions in the kidney [50]. CSE deficiency in mice could lead to reduced renal H_2_S production and increase severity of damage and mortality after renal I/R injury, which indicates that H_2_S may play a role in alleviating renal I/R injury [14]. More recently, there is growing evidence regarding the beneficial effects of H_2_S on ameliorating renal I/R injury mainly via a variety of antioxidant, antiapoptotic, and anti-inflammatory effects (Table 5). These studies indicate that H_2_S and its donors may be of benefit in conditions associated with renal I/R injury, such as renal transplantation.

### 3.4. H_2_S and Cerebral I/R Injury

Ischemic cerebrovascular disease is one of the most common disorders that greatly threaten human health with high morbidity, disability, and mortality [51]. Cerebral I/R injury is mainly characterized by a deterioration of ischemic but potentially salvageable brain tissue of an ischemic injury after reperfusion [52, 53]. There are a number of risk factors involved in cerebral I/R injury, such as excitotoxicity, mitochondrial dysfunction, formation of free radicals, breakdown of the blood-brain barrier (BBB), edema, neuroinflammation, and apoptosis [52–54]. Emerging evidences indicate that H_2_S functions not only as a neuromodulator, but also as a neuroprotectant in the central nervous system [18, 55–57]. In an* in vivo* model of cerebral I/R injury, treatment with low concentration of H_2_S decreased the infarct size and improved the neurological function via antiapoptotic effect, implying that H_2_S has a therapeutic role in cerebral ischemic stroke [18, 57]. DAS, an H_2_S donor, could also protect the brain from I/R injury partly via its antiapoptotic effects [58]. ADT, another H_2_S donor, decreased the infarct size and protected BBB integrity by suppressing local inflammation and nicotinamide adenine dinucleotide phosphate oxidase 4-derived ROS generation [55]. However, it is notable that the effects of H_2_S on cerebral I/R injury are controversial [56]. Treatment with a higher dose of exogenous H_2_S donor could deteriorate the effects of cerebral I/R injury [18, 59]. These opposite effects of H_2_S on cerebral I/R injury may be partially associated with the concentration of H_2_S in brain. This research offers a novel insight for future studies on the cytoprotective effects of a proper dose of H_2_S on central nervous system degenerative diseases, such as Alzheimer's disease and Parkinson's disease.

### 3.5. H_2_S and Intestinal I/R Injury

Intestinal I/R injury is considered to be a major and frequent problem in many clinical conditions, including intestinal mechanical obstruction, abdominal aortic aneurysm surgery, cardiopulmonary bypass, strangulated hernias, liver and intestinal transplantation, mesenteric artery occlusion, shock, and severe trauma [60–64]. This injury can lead to the development of systemic inflammatory response syndrome and multiple organ dysfunction syndrome [62, 63]. Although many advanced treatments have been applied to clinical research, the mortality induced by intestinal I/R injury remains very high [61, 63]. Therefore, it is urgent to develop new therapeutic agents/drugs for the treatment of intestinal I/R injury. Recent studies have shown that H_2_S has anti-ischemic activity in the intestinal I/R model. NaHS could significantly reduce the severity of intestinal I/R injury and dramatically increase the activities of SOD and glutathione peroxidase (GSH-Px) in both serum and intestinal tissue, which suggests that H_2_S protects against intestinal I/R injury by increasing the levels of antioxidant enzymes [63]. In addition, administration of NaHS after the onset of ischemia can attenuate I/R-induced damage of intestinal tissues both* in vitro* and* in vivo* [65]. These observations provide new insight regarding the potential use of H_2_S as a therapeutic agent to limit intestinal I/R injury.

### 3.6. H_2_S and Gastric I/R Injury

Gastric I/R injury is an important and common clinical problem which could lead to mucosal injury [66]. A number of clinical conditions contribute to gastric I/R injury, including peptic ulcer bleeding, vascular rupture or surgery, ischemia gastrointestinal disease, and hemorrhagic shock [66]. However, there are few satisfactory clinical methods in the treatment of gastric I/R injury [67]. H_2_S has been found to play an important role in protecting against gastric I/R injury. Endogenous H_2_S had a protective effect against gastric I/R in rats by enhancing the antioxidant capacity through increasing the contents of GSH and SOD [68]. Another study has shown that NaHS and L-cysteine could protect the gastric mucosa against I/R damage mainly mediated by altering mRNA expression and plasma release of proinflammatory cytokines [69]. Furthermore, NaHS and L-cysteine also showed gastroprotective effects against I/R injury by Keap1 s-sulfhydration, nuclear factor-kappa B dependent anti-inflammation, and mitogen-activated protein kinase dependent antiapoptosis pathway [66]. Thus, H_2_S and its donors may have potential therapeutic value in acute gastric mucosal lesion, which is often caused by I/R.

### 3.7. H_2_S and Hind-Limb I/R Injury

I/R injury can occur in skeletal muscle during elective surgery (i.e., free tissue transfer) and lower extremity arterial occlusion [70, 71]. Limb I/R injury may result in a series of postreperfusion syndromes, such as crush syndrome, compartment syndrome, and myonephropathic-metabolic syndrome [72]. Currently, clinical practice mainly focuses on reducing the duration of ischemia to minimize the ischemic injury in skeletal muscle [70, 71]. Therapeutic interventions that change the biochemical environment during the ischemic and/or reperfusion period may result in amelioration of subsequent cellular damage [71]. Treatment with NaHS for 20 minutes before the onset of hind-limb ischemia or reperfusion could result in significant protection against the cellular damage induced by I/R [71, 73]. However, administration of NaHS for 1 minute before reperfusion did not show any protection against limb I/R Injury [73]. Whether H_2_S could protect against limb I/R injury in a dose- and time-dependent manner needs further investigation.

### 3.8. H_2_S and Lung I/R Injury

Lung I/R injury occurs in various clinical conditions such as lung transplantation, cardiopulmonary bypass, trauma, cardiac bypass surgery, sleeve lobectomy, shock, pulmonary embolism, resuscitation from circulatory arrest, and reexpansion pulmonary edema [16, 74–77]. Lung I/R injury is characterized by increased pulmonary vascular resistance, worsened lung compliance, poor lung oxygenation, edema, and increased pulmonary endothelial permeability [16, 78]. Currently, there is no effective therapy available for the lung I/R injury. The precise mechanism of lung I/R injury needs to be further elucidated [16, 74]. A recent study has shown that preperfusion with H_2_S could attenuate the lung I/R injury by reducing lung oxidative stress [16], which suggests that administration of H_2_S or its donors might be a novel preventive and therapeutic strategy for lung I/R injury.

### 3.9. H_2_S and Retinal I/R Injury

Retinal I/R injury is a common clinical condition and is associated with the loss of neurons, morphological degeneration of the retina, loss of retinal function, and ultimately vision loss [79, 80]. Emerging evidence suggests that retinal I/R injury plays an important role in the pathologic processes of several ocular diseases such as diabetic retinopathy, retinopathy of prematurity, acute glaucoma, and retinal vascular occlusion [81, 82]. Retinal I/R injury often results in visual impairment and blindness because of the lack of effective treatment [81, 83]. One recent study has indicated that rapid preconditioning with inhaled H_2_S can mediate antiapoptotic effects and thus protect the rat retina against I/R injury [84]. ACS67, a H_2_S-releasing derivative of latanoprost acid, possesses neuroprotective properties and could attenuate retinal ischemia* in vivo* and decrease the oxidative insult to RGC-5 cells (retinal ganglion cells)* in vitro* [85]. These results suggest that H_2_S represents a novel and promising therapeutic agent to counteract neuronal injuries in the eye [84]. Further studies are needed to prove the neuroprotective propensity of H_2_S in retinal I/R injury using a postconditioning approach.

## 4. Concluding Remarks

H_2_S is now considered as the third signaling gasotransmitter which plays a broad range of physiological and pathophysiological functions, including vascular relaxation, induction of angiogenesis, regulation of neuronal activity, and glucose homeostatic regulation. H_2_S can be endogenously generated via both enzymatic and nonenzymatic pathways and mainly metabolized through three pathways in mammals. However, whether H_2_S could be generated and metabolized via another pathway should be further studied and confirmed. In addition, more efforts should be made to illuminate the expressions and functions of H_2_S-generating enzymes in different organ and tissue. In order to increase the treatment efficacy of H_2_S, a number of slow-releasing H_2_S donors and H_2_S-releasing hybrid drugs have been successfully designed, synthesized, and proved to be effective* in vitro*,* ex vivo*, and* in vivo*. Novel synthetic strategy should be developed to extend the exposure time of H_2_S donor. Agents/drugs with antiapoptotic, antioxidative, anti-inflammatory, and antitumor effects could be conjugated with H_2_S donor to enhance their therapeutic effects. Furthermore, new drug targeting carrier systems should be designed to effectively transport the H_2_S donor to the targeted organ or tissue.

I/R is a pathological condition that is characterized by an initial deprivation of blood supply to an area or organ followed by the subsequent restoration of perfusion and concomitant reoxygenation. Novel mechanisms associated with I/R need to be further studied and illuminated in addition to the existing pathophysiologic mechanisms. Increasing number of studies have shown that H_2_S could protect against I/R injury in many organs and tissues, such as heart, liver, kidney, brain, intestine, stomach, hind-limb, lung, and retina. Whether H_2_S could exert protection against I/R injury in other organs and/or tissues need to be further demonstrated. In addition, the molecular targets of H_2_S in I/R injury are also needed to be clarified. Ischemic preconditioning, conditioning, and postconditioning are three time frames in the protection against I/R injury. Proper time frame and optimal duration of treatment should be confirmed according to the physicochemical property of H_2_S-releasing compounds. Considering different doses of H_2_S-releasing compounds may exert different therapeutic effects, proper dose range should also be further explored to obtain a better therapeutic efficacy. Currently, researches into the molecular mechanisms of H_2_S in I/R injury using animal experiments have made some progress. Clinical evidence-based research should also be useful in further exploring the little-understood field of the role of H_2_S in I/R injury. In addition, longer-term studies are required to determine whether H_2_S treatment permanently improves organ function following I/R injury and whether this effect reduces long-term morbidity and mortality.

In conclusion, with the rapid developments of design and synthetic strategies, as well as better understanding of the precise mechanisms behind the role of H_2_S in I/R injury, treatment with H_2_S or its donors in proper dose range and time frame will exhibit more potent therapeutic effects against I/R injury in further preclinical research and clinical application.

## Figures and Tables

**Table 1:** The biological characteristics of slow-releasing H_2_S donors.

Compounds	H_2_S release mechanisms	Therapeutic effects	References
GYY4137	Hydrolysis	Vasodilation	[86]
		Anti-inflammation	[19]
		Anticancer	[87]
		Protection of mitochondrial function	[88]
		Regulation of oviductal embryo transport and myometrial contractility	[89, 90]
		Antithrombotic	[91]

ADT	Metabolized by carboxylesterases	Neuroprotection against oxidative stress	[92]
		Protection of blood-brain barrier integrity	[55]

ADT-OH	Metabolized by carboxylesterases	Neuroprotection against oxidative stress	[92]
		Vasorelaxation	[93]
		Antineuroinflammation	[94]

AP39	Metabolized by carboxylesterases	Protection against oxidative mitochondrial DNA damage	[95]

S-Aroylthiooximes	Hydrolysis	Unknown	[96]

S-Propargyl-cysteine	Hydrolysis	Angiogenesis promotion	[97]
		Anticancer	[98]
		Cardioprotection	[99]
		Anti-inflammation	[100]

SG-1002	Activation after oral administration	Cardioprotection	[101]

4-Hydroxythiobenzamide	Hydrolysis	Improvement of wound healing	[102]

Arylthioamides	Thiol activation	Unknown	[103]

N-(benzoylthio)benzamides	Hydrolysis	Unknown	[104]

S-Propyl cysteine	Hydrolysis	Cardioprotection	[99]

*N*-Acetylcysteine	Hydrolysis	Protection against oxidative stress	[105]

*N*-Acetylcysteine ethyl ester	Hydrolysis	Protection against oxidative stress	[105]

SAC^*^	Hydrolysis	Protection against oxidative stress	[99]

PhNCS	Thiol activation	Unknown	[106]

PhNCS-COOH	Thiol activation	Unknown	[106]

Lawesson's reagent	Hydrolysis	Anti-inflammation	[107]
		Protection against gastric damage	[108]

Dithioperoxyanhydrides	Thiol activation	Vasorelaxation	[35]

Thioglycine	Bicarbonate activation	Unknown	[109]

L-Thiovaline	Bicarbonate activation	Unknown	[109]

Thioamino acids	Bicarbonate activation	Vasorelaxation	[109]

Phosphorodithioates	Hydrolysis	Protection against oxidative stress	[35]

S-SH compounds	Thiol activation	Myocardial I/R protection	[110]

N-(acylthio)-benzamides	Thiol activation	Unknown	[104]

H_2_S photo-donor **5**	Light activation	Unknown	[111]

*gem*-Dithiol compounds	Light activation	Unknown	[35]

Allyl isothiocyanate	Thiol activation	Unknown	[112]

Benzyl isothiocyanate	Thiol activation	Unknown	[112]

4-Hydroxybenzyl isothiocyanate	Thiol activation	Unknown	[112]

Erucin	Thiol activation	Unknown	[112]

Sinigrin	Hydrolysis	Unknown	[112]

Poly(ethylene glycol)-ADT	Metabolized by carboxylesterases	Unknown	[113]

S-memantine	Thiol activation	Protection against ischemic neuronal death	[114]

ACS1	Metabolized by carboxylesterases	Neuroprotection	[115]
		Anticancer	[116]

^*^This compound is also a derivative of garlic.

**Table 2:** The biological characteristics of H_2_S-releasing hybrid drugs.

Compounds	Parent drugs	Therapeutic effects	References
ACS2	Valproic acid	Anticancer	[116]
		Antiangiogenesis	[117]

ACS6	Sildenafil	Proerectile	[118]
		Neuroprotection	[119]
		Protection against oxidative stress	[120]

ACS14	Aspirin	Protection against oxidative stress	[121]
		Prevent the progression of atherosclerosis	[122]
		Antiaggregatory	[123]
		Protection against I/R injury	[124]
		Modulation of thiol homeostasis	[125]
		Neuroprotection	[115]

ACS15^*^	Diclofenac	Anticancer	[126]
		Antiosteolysis	[127]
		Anti-inflammation	[128]
		Antiangiogenesis	[117]

ACS18	Sulindac	Anticancer	[126]
		Antiangiogenesis	[117]

ACS21	Salicylic acid	Protection against I/R injury	[124]

ACS32	Diclofenac	Antiosteolysis	[127]

ACS33	Valproic acid	Anticancer	[129]
		Inhibition of histone deacetylase activity	[129]

ACS67	Latanoprost	Regulation of insulin secretion	[114]
		Neuroprotection	[85]

ACS83	L-DOPA	Anti-inflammation	[130]

ACS84	L-DOPA	Anti-inflammation	[131]
		Neuroprotection	[132]

ACS85	L-DOPA	Anti-inflammation	[118]

ACS86	L-DOPA	Anti-inflammation	[118]

ATB-284	Unknown	Prevention against irritable bowel syndrome	[133]

ATB-337^*^	Diclofenac	Anti-inflammation	[134]

ATB-343	Indomethacin	Anti-inflammation	[135]

ATB-345	Naproxen	Anti-inflammation	[136]

ATB-346	Naproxen	Anti-inflammation	[136]
		Anticancer	[137]

ATB-429	Mesalamine	Anti-inflammation	[138]
		Abirritation	[139]

HS-aspirin (HS-ASA)	Aspirin	Anticancer	[140]

Compound **8e**	3-n-Butylphthalide	Antithrombosis	[141]

H2S-EXP 3174	Active metabolite of losartan	Vasorelaxation	[142]

NOSH-aspirin (NBS-1120)	Aspirin	Anticancer	[143]
		Anti-inflammation	[144]

NOSH-naproxen (AVT-219)	Naproxen	Anti-inflammation	[145]

NOSH-sulindac (AVT-18A)	Sulindac	Anti-inflammation	[145]

S-diclofenac^*^	Diclofenac	Anti-inflammation	[146]
		Protection against I/R injury	[146]

S-zofenopril	Zofenopril	Improvement of vascular function	[147]

^*^These compounds are remarkably similar to each other.

**Table 3:** Effects of H_2_S and its donors in myocardial I/R injury.

Experimental models	Effects	Proposed mechanisms	References
Myocardial I/R *in vivo *(rat)	NaHS (0.2 mg/kg, prior to R) protects against the effects of haemorrhage-induced I/R	Upregulation of the protein kinase B/endothelial nitric oxide synthase pathway	[148]

Regional myocardial I/R* in vivo *(rat)	NaHS (3 mg/kg, 15 min prior to I) shows cardioprotective effects	Combination of antiapoptotic and anti-inflammatory effects	[149]

Isolated perfused heart *ex vivo* (rat)	NaHS (100 *μ*M, plus histidine buffer solution, prior to R) enhances cardiac performance	Prevention of apoptosis and preservation of the phosphorylative system	[150]

Isolated perfused heart *ex vivo* (rat)	NaHS (0.1–100 *μ*M, at the onset of R) protects rat heart against I/R injury	Mitochondrial K_ATP_ channel opening	[151]

Primary cultured neonatal cardiomyocytes (rat)	NaHS (25–200 *μ*M, 30 min prior to H) protects cardiomyocytes from oxidative stress	Inhibition of mitochondrial complex IV and enhancement of SOD activity	[152]

Isolated perfused heart *ex vivo* (rat)	NaHS (10 *μ*M, at the onset of R) protects isolated rat hearts from I/R injury	Activation of the Janus kinase 2/signal transducer and activator of transcription 3 signaling pathway	[153]

Isolated perfused heart *ex vivo* (rat)	NaHS (40 *μ*M, throughout the experiment) provides myocardial protection	Possibly activation of the expression of heat shock protein 72	[154]

Isolated perfused heart *ex vivo* (rat)	L-cysteine (0.1–10 mM, 10 min before I until 10 min after R) induces limitation of infarct size	Dependent on H_2_S synthesis	[155]

Myocardial I/R *in vivo *(rat)	NaHS (14 *μ*M/kg, 7 days before myocardial I/R) significantly reduces the myocardial infarct size	Antiapoptotic, antioxidative, and anti-inflammatory activities	[156]

Isolated perfused heart *ex vivo* (rat)	NaHS (100 *μ*M, prior to I) significantly decreases the duration and severity of I/R-induced arrhythmias	Mitochondrial K_ATP_ channel opening	[157]

Isolated perfused heart *ex vivo* (rat)	NaHS (100 *μ*M, prior to I) significantly decreases myocardial infarct size and improves heart contractile function	Activation of K_ATP_/PKC/ERK1/2 and PI3K/Akt pathways	[158]

Isolated cardiac myocytes (rat)	NaHS (100 *μ*M, prior to I) increases cell viability, percentage of rod-shaped cells, and myocyte contractility	K_ATP_/PKC dependent induction of COX-2 expression and nitric oxide-induced COX-2 activation	[159]

Myocardial I/R *in vivo *(mice)	H_2_S (100 ppm, prior to I) has protective properties in I/R injury	Reduction of myocardial ROS production and the inhibition of inflammation, necrosis, and fibrogenesis	[36]

Regional myocardial I/R* in vivo *(pig)	Na_2_S (100 *μ*g/kg bolus + 1 mg/kg/hr infusion, 10 min prior to R) improves myocardial function and reduces infarct size	Anti-inflammatory properties	[160]

Regional myocardial I/R* in vivo *(pig)	Na_2_S (100 *μ*g/kg bolus + 1 mg/kg/hr infusion, throughout the experiment) reduces myocardial infarct size	Antiapoptotic activities	[161]

Regional myocardial I/R* in vivo *(rat)	NaHS (0.1–10 *μ*M, 10 min prior to I until 10 min into R) results in a concentration-dependent limitation of infarct size	Mitochondrial K_ATP_ channel opening	[162]

Myocardial I/R *in vivo *(rat)	NaHS (0.2 mg/kg, prior to R) protects against the effects of haemorrhage-induced I/R	Protection against oxidative stress	[163]

Primary cultured neonatal cardiomyocytes (rat)	NaHS (1–100 *μ*M, 30 min prior to H) shows concentration-dependent inhibitory effects on cardiomyocyte apoptosis induced by H/R	Induction of phosphorylation of GSK-3 and inhibition of mitochondrial permeability transition pore opening	[164]

Myocardial I/R *in vivo *(mice)	Na_2_S (0.1 mg/kg, 7 days prior to I) attenuates myocardial I/R injury	Activation of nuclear factor erythroid-2-related factor-2 signaling in an Erk-dependent manner	[165]

Myocardial I/R *in vivo *(rat)	NaHS (14 *μ*M/kg, 7 days prior to I) inhibits apoptosis of cardiomyocytes induced by myocardial I/R	Enhancement of the phosphorylation of apoptosis repressor with caspase recruitment domain	[166]

Myocardial I/R *in vivo *(mice)	Na_2_S (10–500 *μ*g/kg, prior to R) limits infarct size and preserves left ventricular function	Inhibition of myocardial inflammation and preservation of both mitochondrial structure and function	[167]

Myocardial I/R *in vivo *(mice)	Na_2_S (100 *μ*g/kg, 1 h prior to I) reduces myocardial infarct size	miR-21-dependent attenuation of ischemic and inflammatory injury	[168]

Myocardial I/R *in vivo *(mice)	Na_2_S (100 *μ*g/kg, 24 h prior to I) reduces myocardial infarct size	Combination of antioxidant and antiapoptotic signaling	[169]

Isolated perfused heart *ex vivo* (rabbit)	Allitridum (60 *μ*M, prior to I) reduces myocardial infarct size	Activation of PKC	[170]

Myocardial I/R *in vivo *(mice)	DATS (200 *μ*g/kg, prior to R) significantly reduces infarct size and increases myocardial contractile function	Preservation of endogenous hydrogen sulfide and increase of nitric oxide bioavailability	[32]

Myocardial I/R *in vivo *(mice)	Na_2_S (100 *μ*g/kg, prior to R) protects against the structural and functional deterioration of the left ventricle	Protection against oxidative stress and mitochondrial dysfunction	[15]

Isolated perfused heart *ex vivo* (rat)	NaHS (50 *μ*M, prior or post to I) protects against cardiac I/R injury	Phosphorylation of mammalian target of rapamycin C2	[171]

Myocardial I/R *in vivo *(rat)	NaHS (3 mg/kg, 15 min prior to I) significantly reduces myocardial infarct size	Mitochondrial K_ATP_ channel opening	[172]

Primary cultured neonatal cardiomyocytes (rat)	NaHS (30 *μ*M, 30 min prior to H) attenuates cardiomyocyte apoptosis and enhances cell viability	Protection of cardiomyocytes against I/R-induced apoptosis by stimulating Bcl-2	[173]

Isolated perfused heart *ex vivo* (mice)	Na_2_S (10 *μ*M, 40 seconds after the start of R) markedly improves the recovery of myocardial function	Nitric oxide synthase 3-dependent signaling pathway	[174]

Myocardial I/R *in vivo *(rat)	NaHS (14 *μ*M/kg/d, 6 d prior to I) markedly reduces heart infarct size and has great improvement in blood pressure	Upregulation of survivin	[175]

Myocardial I/R *in vivo *(pig)	NaHS (0.2 mg/kg, prior to R) markedly reduces myocardial infarct size and improves regional left ventricular function	Higher expression of phospho-GSK-3*β* and lower expression of apoptosis-inducing factor	[176]

H/R: hypoxia/reoxygenation; SOD: superoxide dismutase; PKC: protein kinase C; ERK1/2: extracellular signal regulated kinase 1/2; PI3K (PtdIns3K): phosphatidylinositol 3-kinase; Akt (PKB): protein kinase B; COX-2: cyclooxygenase-2; ROS: reactive oxygen species; GSK-3: glycogen synthase kinase-3.

**Table 4:** Effects of H_2_S and its donors in hepatic I/R injury.

Experimental models	Effects	Proposed mechanisms	References
Hepatic I/R *in vivo *(rat)	NaHS (28 *μ*M/kg, prior to R) attenuates the injured hepatic function and the synthetic action of hepatocytes	Inhibition of lipid peroxidation and inflammation reactions	[177]

Hepatic I/R *in vivo *(mice)	NaHS (1.5 mg/kg, 1 h prior to I) protects against hepatic I/R injuries	Activation of the PtdIns3K-AKT1 pathway	[17]

Hepatic I/R *in vivo *(rat)	NaHS (14 *μ*M/kg, 30 min prior to I) significantly attenuates the severity of liver injury and inhibits the production of lipid peroxidation	Antioxidant and antiapoptotic activities	[46]

Hepatic I/R *in vivo *(rat)	DAS (1.75 mM/kg, 12–15 h prior to I) protects the liver from warm I/R injury	Induction of heme oxygenase-1 and inhibition of cytochrome P450 2E1	[178]

Hepatic I/R *in vivo *(mice)	Na_2_S (1 mg/kg, 5 min prior to R) protects the murine liver against I/R injury	Upregulation of intracellular antioxidant and antiapoptotic signaling pathways	[179]

Hepatic I/R *in vivo *(mice)	H_2_S (100 ppm, 5 min prior to R) protects the liver against I/R injury	Reduction of necrosis, apoptosis, and inflammation	[180]

Hepatic I/R *in vivo *(mice)	NaHS (14 and 28 *μ*M/kg, 30 min prior to I) attenuates hepatic I/R injury	Weaken the apoptosis through the inhibition of c-Jun N-terminal protein kinase 1 signaling pathway	[181]

Hepatic I/R *in vivo *(rat)	NaHS (12.5, 25 and −50 *μ*M/kg, 5 min prior to I) reduces liver damage after perioperative I/R injury	Inhibition of mitochondrial permeability transition pore opening, reduction of cell apoptosis, and activation of Akt-GSK-3*β* signaling	[182]

**Table 5:** Effects of H_2_S and its donors in renal I/R injury.

Experimental models	Effects	Proposed mechanisms	References
Renal I/R *in vivo *(mice)	NaHS (1 mg/kg, 15 min prior to I) rescues mice from the injury and mortality	Modulation of oxidative stress	[14]

Renal I/R *in vivo *(mice)	H_2_S (100 ppm, before and after treatment) shows protective effects on survival, renal function, apoptosis, and inflammation	A hypometabolic state induced by H_2_S	[183]

Renal I/R *in vivo *(pig)	Na_2_S (100 *µ*g/kg, 10 min prior to R) results in a marked reduction in kidney injury and preserves glomerular function	Anti-inflammatory effects	[184]

Isolated perfused kidney *ex vivo* (pig)	H_2_S (0.5 mM, 10 min before and after R) ameliorates the renal dysfunction	Activation of K_ATP_ channels	[185]

Renal I/R *in vivo *(mice)	NaHS (100 *µ*M/kg, 30 min prior to I) significantly attenuates I/R injury-induced renal dysfunction	The increase in expression of CSE	[186]

Renal I/R *in vivo *(rat)	NaHS (100 *µ*M/kg, 15 min prior to I and 5 min prior to R) attenuates renal I/R injury	Antiapoptotic and anti-inflammatory effects	[187]

Warm renal I/R *in vivo *(rat)	NaHS (150 *µ*M, at time of renal pedicle clamping and during R) improves long-term renal function and decreases long-term inflammation	Antiapoptotic and anti-inflammatory effects	[188]

Warm renal I/R *in vivo *(rat)	NaHS (150 *µ*M, during I and R) increases renal capillary perfusion and improves acute tubular necrosis and apoptosis	Decrease of leukocyte migration and inflammatory responses	[189]

Renal I/R *in vivo *(pig)	Na_2_S (2 mg/kg, 2 h prior to I) attenuates tissue injury and organ dysfunction	Antioxidant and anti-inflammatory effects	[190]

Renal I/R *in vivo *(rat)	NaHS (100 *µ*g/kg, 20 min prior to I or 10 min prior to R) protects against renal I/R injury	Antioxidant and antiapoptotic effects	[191]
